# Laser-Enhanced Machine Vision for Edge Profile Measurement of Thin Film Printed Electronics

**DOI:** 10.3390/mi17080964

**Published:** 2026-08-15

**Authors:** Mothana A. Hassan, Ali Abdulkhaleq Alwahib

**Affiliations:** College of Laser and Optoelectronics Engineering, University of Technology-Iraq, Baghdad 10066, Iraq; ali.a.alwahib@uotechnology.edu.iq

**Keywords:** laser-based machine vision, laser light source, surface metrology, thin film edge profiling, image processing, non-contact optical inspection

## Abstract

Thin film printed electronics, such as flexible circuits and sensor sheets, require non-contact inspection to detect defects and edge degradation. The present paper presents a laser-enhanced machine vision framework for detecting and analyzing the edges of printed conductive tracks using Canny edge detection and Otsu thresholding. Using a coherent laser source, Otsu’s method enhances contrast at the ink–substrate interface, enabling robust segmentation of edge lines. Canny operator is applied to thresholded images to extract precise edge profiles. Multiple printed tracks are analyzed to calculate four lateral edge roughness values (*Ra*). As a result, the values are 40.43 µm, 40.09 µm, 50.26 µm and 40.94 µm. The results show that the suggested method can detect and qualify variations in edge parameters. Printed electronics are produced using an inline inspection and quality control system based on non-contact, high-resolution, and scalable technologies.

## 1. Introduction

Innovating technologies such as flexible displays, wearable sensors, and photovoltaic energy systems heavily rely on thin film electronics. Flexible displays, wearable seniors, and photovoltaic systems require reliable inspection and process control of this film’s functional layers. There are various types of surface anomalies, such as cracks, pinholes, scratches, and delamination’s, which can affect electronic performance, optical transparency, or mechanical stability. As a result, it is important to detect and classify these defects at the time of fabrication as well as afterwards. There are many traditional metrology techniques, including scanning electron microscopy (SEM), ellipsometry, and stylus profilometry. The limitations of these methods, however, must be considered. Particularly when measuring features that range from hundreds of nanometers to micrometers in size, the resulting measurements may differ from those obtained using more established techniques, such as ellipsometry. Inline inspection is not possible with stylus profilometers due to their contact-based design and potential to damage fragile films. In addition to measuring thickness accurately, ellipsometry is ineffective at locating defects. To image at the nanometer scale, SEMs are real-time imaging techniques that are used to observe the dynamical process at the nanoscale range, but are destructive, expensive, and slow techniques. Optical non-contact inspections therefore provide a better means of detecting defects in situ in real-time [1,2,3,4].

Laser-based optical systems, particularly those using coherent light sources, have enabled the resolution of subwavelength anomalies. It has been shown that by using interferometry, a nanometer-scale interference pattern can be produced from surface irregularities. Custom interferometric analysis software revealed nanometer-scale surface topography and confirmed the capability of the system to provide broadband detection across the wavelength range of the light source [5,6,7,8]. Interferometric systems, however, have some challenges, including vibration sensitivity, alignment precision, and complicated interpretations. A combination of robust image processing algorithms and optical machine vision (MV) techniques has been demonstrated to offer advantages when it comes to simplicity, cost efficiency, and speed [9,10,11]. When it comes to defect detection in MV, a hybrid approach is adopted that integrates the Otsu thresholding algorithm that emphasizes intensity gradients for delineating object boundaries in conjunction with the Canny edge detection method. It is an automatic global thresholding method for image segmentation. Several thin film solar cell and printed electronics defect detection techniques have demonstrated excellent performance [12,13,14]. Otsu’s algorithm minimizes intra-class intensity variance to identify an optimal threshold while Canny filtering identifies discontinuities by convolving an image with derivative kernels [15,16,17].

Recently, MV systems have been applied successfully to extract surface edge profiles from printed films using optical images under white light illumination as previously reported [18]. Using calibrated pixel-to-height translation from optical microscopy, this non-contact approach eliminates the need for SEM while providing sensitive edge characterization. By utilizing their method, defects can be monitored inline with minimal equipment overhead, and hybrid optical inspection is made viable. As an extension of this approach, we propose a laser-based illumination system that could enable greater coherence and enhanced sensitivity to defects at the microscale. Monochromatic lasers are helpful in enhancing surface scattering and reflectance contrast, which is helpful in improving edge detection performance. As a result of this modification, printed electronic devices can be inspected in real time, particularly for films thicker than 20 nm that require precise lateral edge roughness measurements [19,20,21]. These tools take advantage of open-source platforms such as MATLAB, R2023b incorporating pre-processing (grayscale conversion, denoising), segmentation (Otsu thresholding), and edge feature extraction (Sobel, Laplacian, and Canny). Furthermore, blob detection and contour analysis were used for quantifying defect area, perimeter, and shape descriptors, able to distinguish dust particles, short circuits, or delamination’s in films [22,23,24]. It has become increasingly common to use MV in surface metrology, offering a robust alternative to traditional profilometry and microscopy. It is now possible to extract areal surface parameters such as roughness, step height, and edge profile geometry accurately using MV systems, especially when combined with calibrated optical systems. This technique uses high-resolution 2D images to reconstruct 3D surface maps using photometric stereo or structure-from-motion algorithms, enabling the non-contact analysis of microscale topographies. MV-based metrology is especially useful in roll-to-roll (R2R) manufacturing, flexible electronics, and other applications where traditional methods are challenged by speed, resolution, or destructive sampling [25,26,27].

In this article, we propose a fully optical, laser-enhanced MV approach for the detection of edge profile degradation and surface defects in thin flexible printed electronic structures. This approach can be used to demonstrate the efficiency of thin film quality assurance for research and industrial thin film applications without requiring expensive SEM or profilometry. Laser-enhancing MV is presented here to detect deterioration of edge profiles in thin printed films and surface defects. In this study, the objective is to demonstrate how effective this approach might be in addressing thin film quality measurements in research and industry as a rapid, low-cost alternative to SEM and profilometry as a means of measuring thin film quality.

This work aims to develop and validate a laser-enhanced MV framework for measuring edge profiles on printed electronic structures. In MV applications, laser illumination is commonly used due to its well-established optical characteristics, including spatial coherence, monochromaticity, and directional illumination. To validate the proposed inspection framework, the study focused on validating the proposed inspection framework rather than evaluating different illumination sources.

In addition, a HeNe laser operating at 650 nm improves spatial coherence and speckle contrast. Conductive silver ink is being investigated for a wide variety of applications in different fields. Silver-based conductive ink can be imaged on flexible substrates with this technique. Consequently, the MV algorithms required a stronger backscattered signal at material boundaries to detect edges accurately. This is due to the contrasts in refractive indices between the materials at their boundaries. In combination with improved lighting geometry and machine learning enhancements, sub-visible changes in edge sharpness and continuity can be detected before they cause functional problems. For a machine vision algorithm to perform correctly, there needs to be a stronger backscatter signal present at the borders of the material in order to compensate for the greater contrast between the refractive indices of the materials at the boundaries. The goal of this project is to develop improvements in lighting geometry as well as machine learning enhancements so that small changes in edge sharpness or film continuity can be identified prior to causing functional failures in the system due to functional failures caused by these changes.

As printed electronics evolve in complexity and miniaturization, lasers are proving to be an increasingly valuable instrument for improving MV. It is particularly timely to combine coherent laser illumination with advanced MV techniques due to the complexity of printed electronics and the miniaturization of electronics. With its high performance, non-destructive capabilities, and automation-friendly features, this technology is suitable for photovoltaics, biosensors, and flexible circuit manufacturing.

## 2. Flexible Printed Electronic Measurement Sample Type

**Figure 1:**
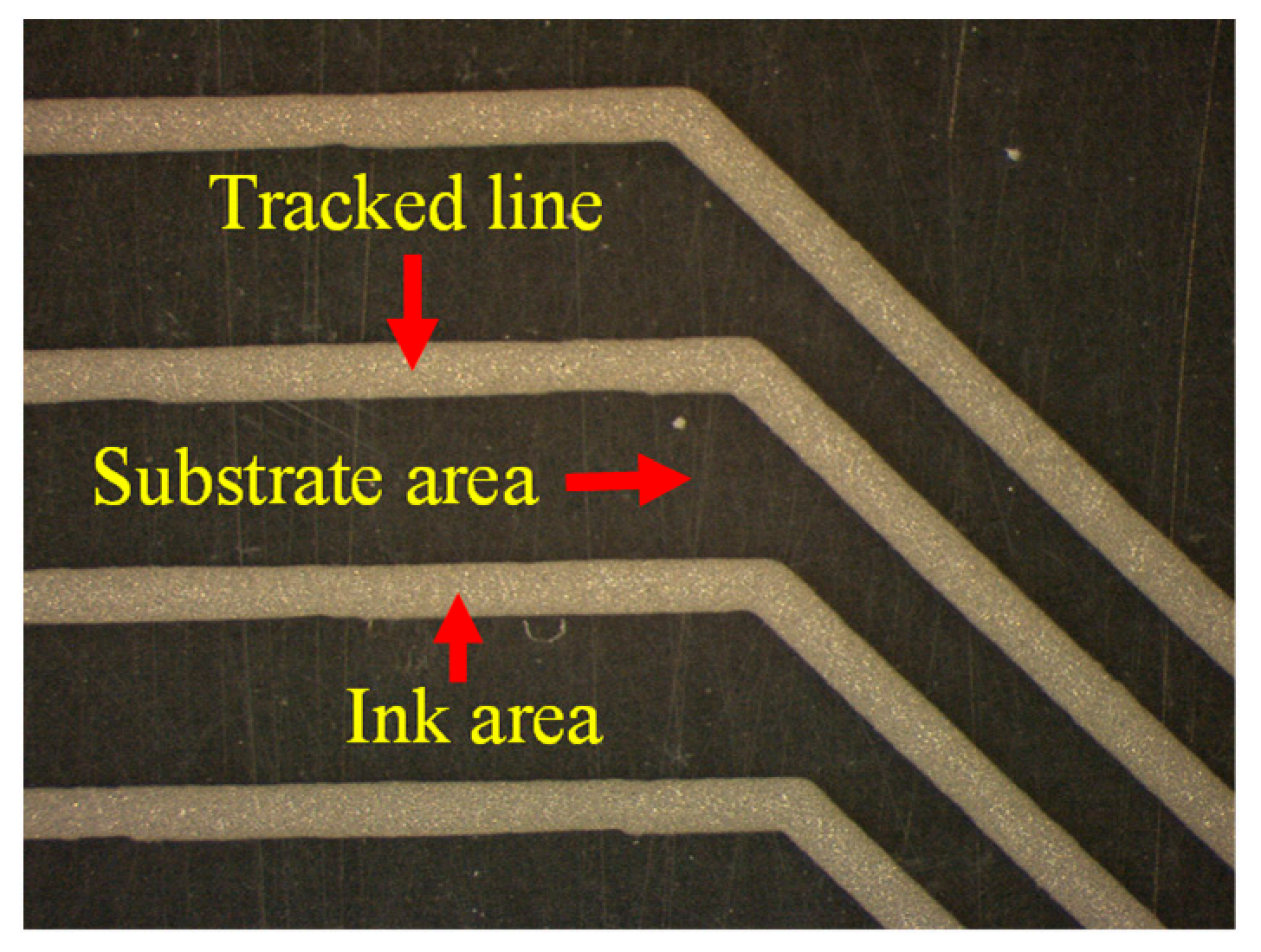
PC keyboard’s flexible thin film printed electronic circuit sample.

As an experimental sample, a PC keyboard’s flexible membrane circuit was used. On a PET substrate, the sample represents a thin conductive layer with an approximate thickness of 20 μm. The printed conductive tracks have an approximate width of 2 mm. Silver-based ink is used for conductive materials, while the exact printing process is used to manufacture the commercial keyboard membrane in the screen-printing method as shown in Figure 1. This type of ink associations silver flakes or nanoparticles with a polymer matrix, providing excellent electrical conductivity and compatibility with flexible surfaces, such as polyethylene terephthalate (PET). The mechanical flexibility, durability, and affordability of PET have made it a popular material. Screen printing and flexography are well-suited to silver ink because of its excellent adhesion to PET surfaces and low processing temperature. Moreover, carbon-based inks may be used in certain circuit areas to increase wear resistance, such as contact pads. Furthermore, dielectric inks are often used in multilayered structures as insulating layers. By layering these circuits, it is possible to maintain low manufacturing costs while integrating complex circuits for modern keyboards.

## 3. Experimental Setup

Through the use of a fully optical, non-contact MV system, in this experiment, thin flexible printed electronic structures were evaluated for edge quality and defect characteristics. Using an integrated laser illumination module and a high-resolution imaging camera, edge-enhanced profiles of coated films were captured. To enable MV algorithms to work properly, the setup was optimized for real-time inspections, enabling high-contrast imaging.

**Figure 2:**
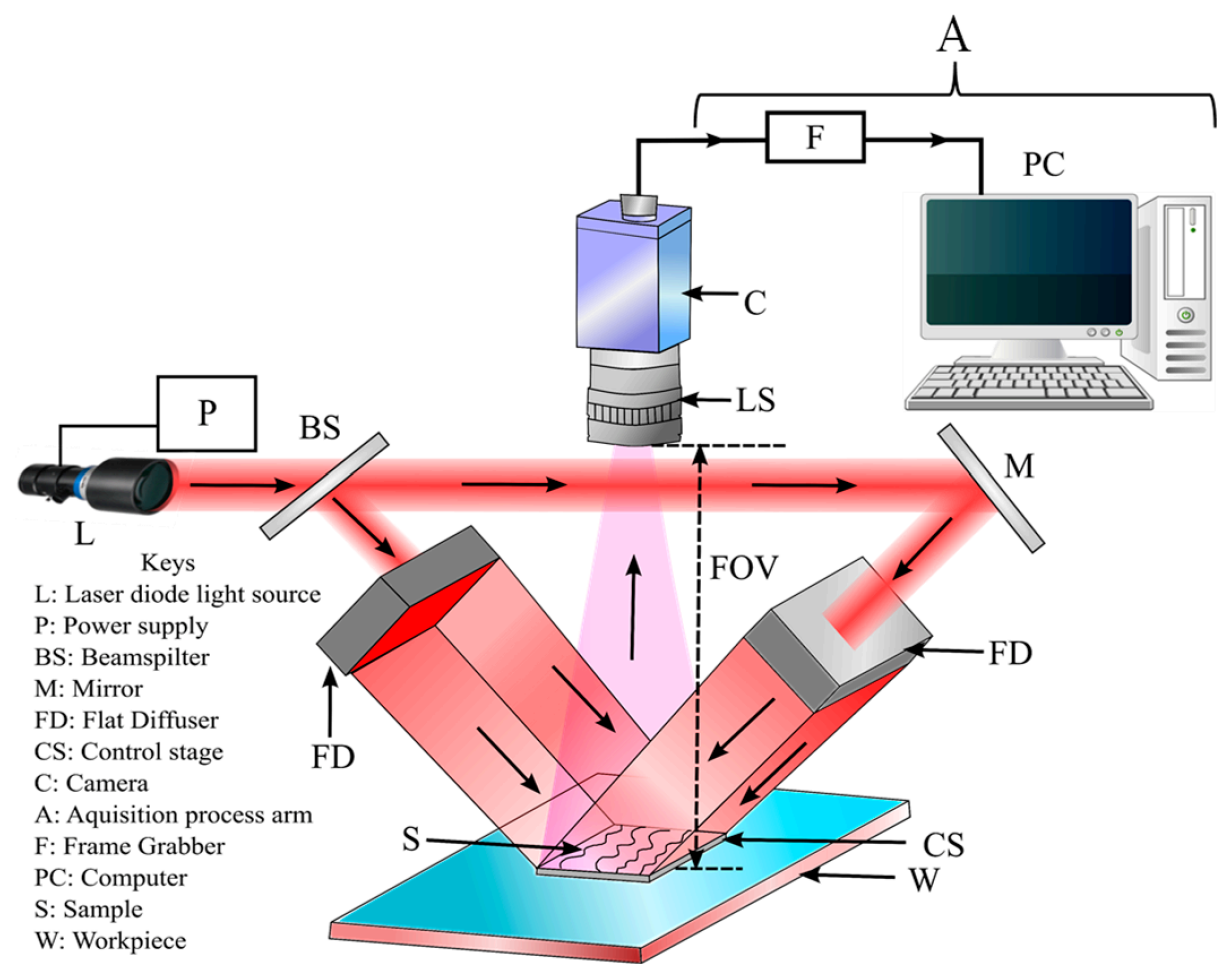
Schematic diagram of MV system integrated with laser light source, flat diffuser, sample (thin film), and high-resolution camera connected with PC.

A careful calibration was conducted on each subsystem to ensure sensitivity to surface variations at the microscale without physical contact (Figure 2). A MV system is installed to the external laser light that illuminates a target sample surface.

### 3.1. Laser Illumination Subsystem

A laser illumination subsystem as a light source is the heart of the optical MV setup used for non-contact defect detection in printed thin films. In this study, a continuous-wave (CW) diode laser (L650P007, 650 nm, 7 mW, Ø5.6 mm, A Pin Code, Laser Diode, Thorlabs, Newton, NJ, USA), operating at 650 nm (visible), was used because of its stable output and effective interaction with the thin film surface. A wavelength of 650 nm was selected to improve the contrast of edge defects, thanks to surface scattering. Through backscattered signal intensities, the inspection system enhances contrast at material boundaries. In particular, coherent illumination is sensitive to geometric discontinuities, which makes micro-defects along printed track edges more visible. As coherent sources can interfere with surface microstructures and result in speckle effects, a diffusing element has been incorporated into the illumination path to spatially randomize the phase distribution of the beam, thus providing a uniform illumination field at the sample. As a result of this configuration, speckle contrast is reduced, and intensity-based segmentation methods are more stable.

This was based on the optical properties of the film and the type of defect targeted. A careful adjustment of the laser power was made to ensure adequate illumination without damaging the delicate coating. It was necessary to ensure a well-defined and uniform spot using TEM00 Gaussian beam profile. A beam expander and collimating lens were used to optimize the beam diameter and divergence for uniform coverage. For increased signal clarity, bandpass filters centered on the laser wavelength were placed in front of the imaging sensor. A diffuser or polarizer was added to minimize glare and enhance feature visibility, respectively, by modifying light scattering and polarization. Using carefully shaped and filtered beams, high-contrast images were captured by a CMOS camera, which were then processed with MV algorithms to detect edges and defects. It is crucial to position the laser correctly with respect to both the sample and the camera so that defect visibility can be improved, and it depends on how the optical interaction should be conducted. Using the laser, the camera is aligned to capture the specular reflection on the sample surface.

In this setup, surface defects such as scratches, dust, and irregular roughness can be highlighted most effectively. It is possible for the detection geometry as well as the incident angle of the system to be adjusted to achieve a higher contrast between defective and non-defective regions, thus improving the performance of the system. Lighting plays a major role in the design and performance of machine vision (MV) systems. There is no doubt that lasers are becoming more important for precision-oriented medical imaging in the coming years due to their uniform spectral content despite the uniform coverage of white light sources and their broad spectral content. To achieve tasks such as high spatial resolution, depth sensing, and directional illumination, lasers are ideal because they offer unique optical characteristics such as a high coherence level, monochromaticity level, and directionality level. Several applications, including 3D surface profiling, defect detection, and dimensional metrology, as well as automated microstructure inspection, are better served by laser-based systems than by white light alternatives. By generating focused, high-intensity beams, they can project precise patterns (e.g., laser lines, dots, or planes) suitable for triangulation-based 3D vision, laser scanning, and interferometric surface measurements thanks to their ability to generate a focused, high-intensity beam. Laser speckle effects can also be exploited to detect submicron surface deviations through speckle interferometry and phase-shift analysis, which are often ignored in conventional imaging. In contrast, white light sources are more suited for applications requiring color analysis, area-wide illumination, or high lighting uniformity, such as label recognition, barcode scanning, and presence/absence detection, that require color analysis, illumination across a wide area, or high lighting uniformity. While they are suited to applications such as structured light and high-precision metrology, they lack spatial coherence and a broad spectral content [28]. The purpose of Table 1, as well as the accompanying literature, was to justify the choice of laser illumination for this proposed inspection system as opposed to presenting an experimental comparison between different sources of illumination [29,30].

In Table 1, established characteristics reported in the literature are summarized and used to motivate the selection of laser illumination for the proposed system. Nonetheless, it should be interpreted as a comparison performed by this study as opposed to the previous studies.

### 3.2. Flat-Top Holographic Diffuser Sets in Machine Vision Applications

Laser-based MV systems need uniform illumination across the field of view to acquire precise images and analyze surfaces. Laser beams with a Gaussian intensity profile, on the other hand, suffer from a gradual decline in brightness as they move towards the edges. The non-uniform illumination can make a variety of tasks more challenging, including surface inspection, feature recognition, and metrological analysis. These factors may lead to image distortion, overexposure, and inconsistent results. A Flat-Top Holographic Diffuser (FTHD) (Large Angle Flat-Top Holographic Diffuser Set, C:11, Square, Edmund Optics, Barrington, NJ, USA) has been used in current experiments to improve laser beam focus by reshaping the intensity distribution of the laser beam into a flat surface. To maximize contrast and balance illumination, flat diffuser elements were placed on both sides of the sample under test. FTHD changes the angle distribution of transmitted light, redistributing the Gaussian laser beam to produce a more uniform illumination profile.

This method results in improved imaging quality and MV measurements by providing even glare-free illumination. Aside from the coherence, the directionality, and the narrow bandwidth of the FTHD, there are also no artifacts that are associated with Gaussian beam profiles. In surface metrology, where speckle patterns must be uniform, and structured lighting applications, where depth distortion can be eliminated, flat illumination is advantageous. The diffusers that are available can be configured in a variety of shapes, including circular, square, or rectangular, and can be optimized for output angles ranging from 1° to 60°, depending on the application for which they are being used. The liquid crystals are made from high-quality materials, such as optical polymers and borosilicate glass, and are designed to accommodate popular laser wavelengths, such as 405 nm, 532 nm, 650 nm, 800 nm, and 1064 nm. Despite their advanced beam-shaping ability, these diffusers offer high transmission efficiency, often exceeding 80%, ensuring that optical power is lost at its lowest level. MV and optical metrology have become increasingly important due to the use of FTHD sets. As a result of providing uniform illumination across camera sensors, laser-based machine vision systems are primarily used to improve edge recognition, form recognition, and contrast. Flat-top illumination enables accurate detection of defects when inspecting surfaces with highly reflective or irregular surfaces. For optical metrology, diffusers produce a spatially uniform speckle pattern that can be used for quantitative analysis, particularly phase-shift interferometry and laser triangulation. Additionally, they help eliminate intensity fall-off and hotspots that could degrade depth information in 3D imaging and structured light scanning systems. A similar approach can be applied to digital holography and laser microscopy, where even illumination improves resolution and reduces artifacts. In general, FTHD sets improve the overall fidelity and repeatability of visual inspection and metrological processes by improving lighting uniformity, reducing optical noise, and improving fidelity. Due to their high performance, they are an indispensable component of optical sensing and imaging systems of the modern era.

### 3.3. Sample Handling and Positioning

In order to ensure consistent and reliable imaging during the inspection process, the printed electronic thin film samples were mounted on a precision-controlled stage to ensure a high level of accuracy. This stage was designed to allow fine adjustments in the X and Y axes to allow for accurate scanning across the surface of the sample, while the optional *Z*-axis control allowed for optimal focus in relation to the optical path of the sample. To accomplish the movement, a piezoelectric transducer (PZT) actuator (Piezo Step Linear Motor Actuators Type N-310 NEXACT-PiezoWalk, Physik Instrumente, Karlsruhe, Germany) provided extremely precise positioning with micrometer-level accuracy. To control PZT movement for XY stage, LabView 2021 software has been used for this method. During the inspection of optical components and samples, a rigid mechanical stage was employed to minimize mechanical vibration. Flexible films are mounted securely and freed from warping or tension-induced artifacts using a secure mounting mechanism. The predetermined sample characteristics dictated whether a vacuum chuck or soft-edge mechanical clamps should be used to maintain uniform contact without damaging the substrate. By ensuring repeatability of the measurement conditions, and by capturing high-quality data for MV analysis, high-quality data could be captured for subsequent analysis.

### 3.4. Machine Vision Sensor

An imaging system (Kiralux 5.0 MP Color CMOS, Camera, CS505CU, Thorlabs, Newton, NJ, USA) with high-resolution color imaging was used to capture detailed images of the printed thin film (sample under test). Consequently, the inspection could be performed with a reduced impact of color artifacts. In order to meet the inspection task’s spatial resolution and acquisition requirements, cameras with different sensor sizes, resolutions, and frame rates were selected. Fast image acquisition and processing are enabled by GigE or USB 3.0 interfaces, which provide high-bandwidth data transfer between the camera and the image acquisition unit. The magnifying lens has been chosen with respect to camera specifications (magnifying lens 1.5×, MVL6X15L, Thorlabs, Newton, NJ, USA). A Kiralux CS505CU CMOS camera is utilized for the imaging system, with a sensor resolution of 2448 × 2048 pixels and a pixel size of 3.45 μm. The imaging system utilized a 1.5× magnifying lens. The CS505CU camera can capture up to 53.2 frames/s at 2448 × 2048 pixels. To minimize motion related image blur while maintaining sufficient contrast, an exposure time of 30 µs was used in the present experiment. This study did not evaluate the complete acquisition, data transfer, and image processing times of the proposed system; therefore, the acquisition rate should not be construed as the overall inspection or processing rate, as the present study did not measure MATLAB processing time or system throughput experimentally. The spatial sampling resolution is approximately 2.30 μm per pixel using the optical configuration used in the setup. Typically, it is required that there be two or three pixels for each structural feature to detect it reliably, according to the Nyquist sampling criteria. As a result, the proposed system detects defects of approximately 4.6 to 6.9 µm in size, allowing for the identification of microscale defects in printed conductive tracks. We describe the mathematical methods and reasoning used to assess the resolution and imaging performance of a system, including its system resolution, detection limit for defects and field of view (FOV) in this paper.

- Object space sampling resolution is limited by pixels:

The space sampling resolution occurs as a function of the pixel size and magnification in most MV inspection systems, including in a macro inspection setup with 1.5× imaging magnification.

Therefore, the object space sampling of MV system = 3.45/1.5 = 2.30 µm/pixel.

- Detection limit for defects:

It is necessary to cover 2–3 pixels to detect a feature reliably, as per the Nyquist sampling method.

Minimum defect size = 2–3 × 2.30 µm = 4.60–6.9 µm

The MV system can be detected = 4.6–6.7 µm of sample feature size.

- Field of view (FOV) of the MV system:

In the case of a full sensor, the horizontal FOV:

2448 × 2.30 = 5.63 mm

and the vertical FOV:

2048 × 2.30 = 4.71 mm

The full imaging area = 5.63 mm × 4.71 mm. As a result, suitable optics have been chosen to allow a clear visualization of fine defects at the magnification and working distance required to clearly identify them. For applications requiring precise dimensional analysis, a telecentric lens was considered to reduce perspective errors. The aperture was adjusted to optimize both the light intensity and depth of field. Image acquisition and processing were managed using a dedicated computer system, with optional use of a frame grabber if needed for the interface. Software tools ThorImageCAM V1.0.7 software (Thorlabs, USA) were used to control the camera settings and perform initial image capture and processing (see Figure 2). We acquired 25 images under identical experimental conditions to assess the stability and consistency of our proposed MV system. An edge profile roughness (*Ra*) analysis was conducted on one representative image after confirming that the acquired images exhibited consistent edge characteristics.

## 4. Functions for Image Analysis (Image Processing Steps)

**Figure 3:**
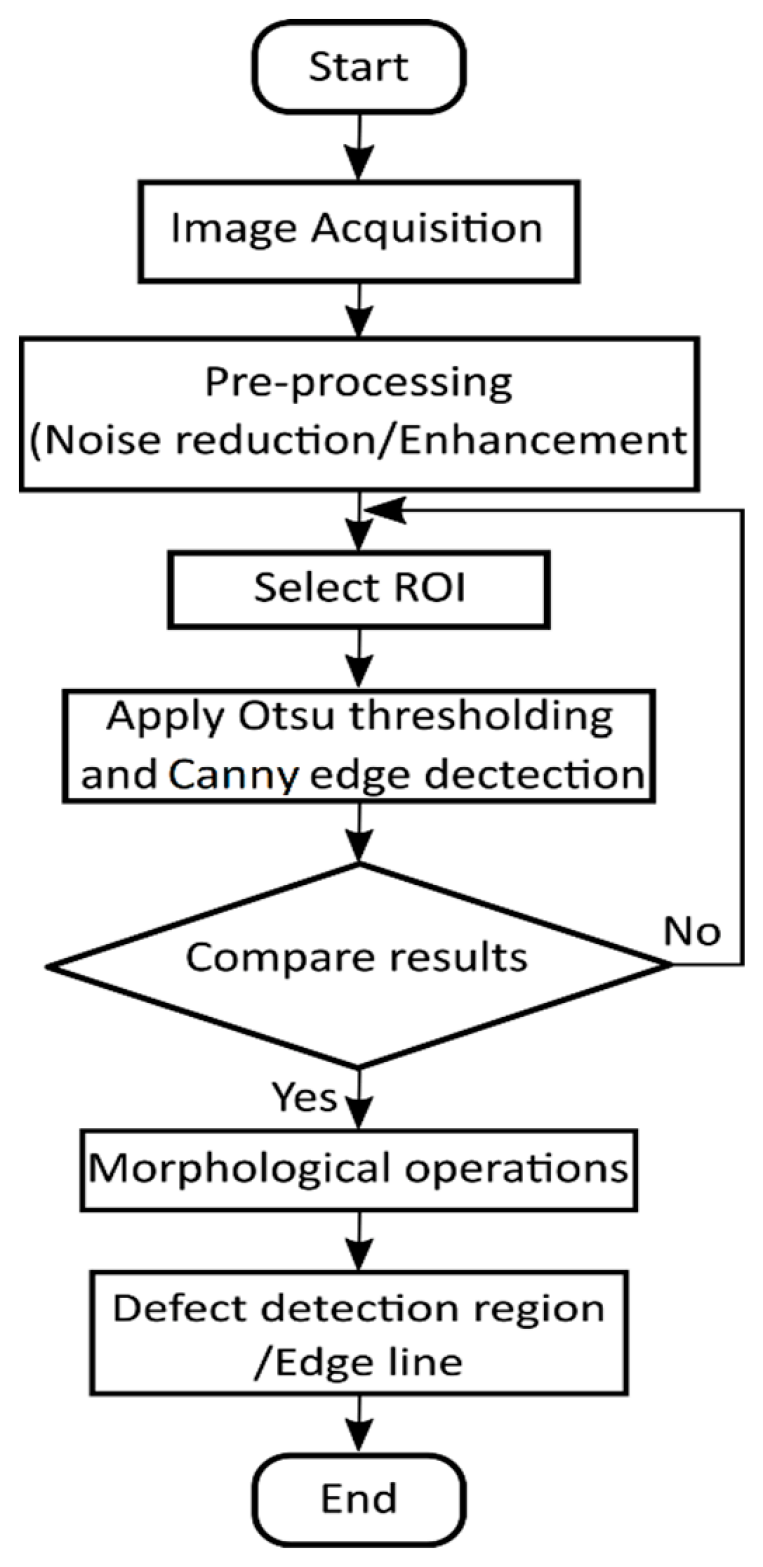
Overall controlled flowchart of MV system for surface inspection.

Functions were used to define and extract specific areas of the image for analysis, focusing on the sample area (ROI). The operating system flowchart is illustrated as shown in Figure 3.

### 4.1. Otsu Thresholding and Canny Edge Detection Analysis

Within the realm of MV approach, image segmentation and edge detection are fundamental steps in identifying and characterizing defects. In terms of automatic thresholding techniques, the Otsu method stands out as one of the most effective and widely used. This enables the algorithm to determine the optimal threshold value by maximizing the inter-class variance of the pixel intensities in the image, thus effectively separating the foreground (potential defects) from the background [15]. An image containing a bimodal histogram, as is often the case when defects exhibit different optical properties from defect-free substrates, is particularly useful for the present method.

The method for selecting the segmentation threshold is based on maximization of the between-class variance. The algorithm assumes that there are two classes (object pixels and background pixels) and then finds the optimum threshold (*T*) for the one-dimensional grayscale image, to minimize the weighted sum of variances of the two classes. To get the final threshold *T*, the method must go through the following steps. Given a grayscale image, the probability of the gray level, the mean gray level, and the total mean gray level can be calculated. In these equations, the image size is defined as *m* × *n* and the number of pixels of the gray level can be determined. For histogram normalization, it can represent it as [15]:

(1) $$p(i)=\frac{n_{i}}{N},\;\;\;i=0,2,\ldots ,L-1$$

where $n_{i}$ = number of pixels at intensity *i*, *N* = total number of pixels, and *L* is the number intensity levels (example: 256 for 8-bit grayscale). For class 1: pixels with intensity values ≤ *T*, and for class 2: pixels with intensity values > *T* (Equation (2)):

(2) $$\omega_{o}(T)=\sum_{i=0}^{T}p(i),\;\;\omega_{1}(T)=\sum_{i=T+1}^{L-1}p(i)$$

where threshold (*T*): 1. $\omega_{o}(T)$ at class 1 probability and 2. $\omega_{1}(T)$ at class 2 probability. Alternatively, it can be computing class means (class 1 and 2 respectively) as:

(3) $$\mu_{o}(T)=\frac{1}{\omega_{o}(T)}\sum_{i=0}^{T}i\cdot p(i),\;\;\mu_{1}(T)=\frac{1}{\omega_{1}(T)}\sum_{i=T+1}^{L-1}i\cdot p(i)\;$$

where the global mean can be calculated as set in Equation (4):

(4) $$\mu T=\sum_{i=0}^{L-1}i\cdot p(i)\;$$

The between-class variance can be computed as:

(5) $$\delta_{b}^{2}(T)=\omega_{0}(T)\cdot \omega_{1}(T)\cdot {\left[\mu_{0}(T)-\mu_{1}(T)\right]}^{2}$$

**Figure 4:**
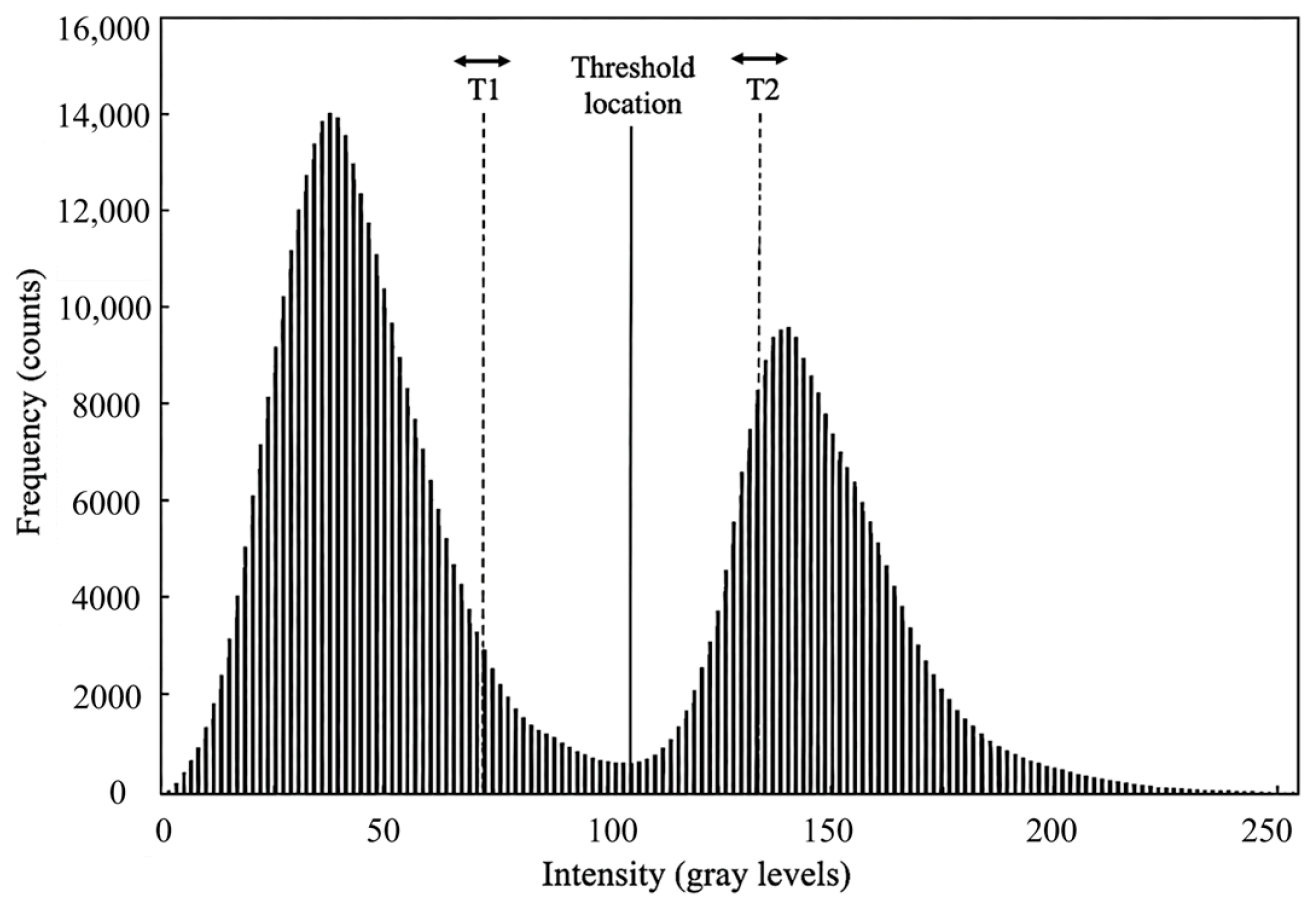
Otsu’s threshold selection method is based on the intensity histogram of the captured image. Pixel intensity levels are represented on the horizontal axis, while pixel frequency is represented on the vertical axis.

Final calculation is to find *T*, that maximizes between-class variance as:

(6) $$T^{*}=\arg\max_{T}\delta_{b}^{2}(T)$$

where $T^{*}$ is the optimal threshold. The gray level histogram of gray image can be defined as an array of size $L\left(0\leq i<256\right)$. In the (*x*, *y*) coordinate, the gray level *i* is histogram (*i*) and it indicates how many pixels of the gray level. Then, the grayscale histogram can be calculated. *R*(*i*) is the computed histogram of grayscale image. Usually in segmentation applications, the observed gray levels of object and background pixels are assumed to be identically distributed and the classes to be identified are unknown. The optimal segmentation threshold *T* by maximization of the between-class variance in two-class problems can be formulated to a minimization of the within-class variance: where the optimum threshold is T* corresponding to the point that maximizes J (*T*); where *L* is the number of possible gray levels, *Pi* is the probability of gray level *i*. Let *L* = 256, max no. (*i*) = 255. From the histogram, the probability of different gray levels can be calculated as follows: $P\left(i\right)\;=\;pd\left(i\right)/\left(m\times n\right)$. Using max. J(*i*), *T* is the threshold. If an image is pre-processed using two-dimensional histogram equalization, the histogram of gray image shifts to the normal distribution, then the threshold is optimum (see Figure 4).

**Figure 5:**
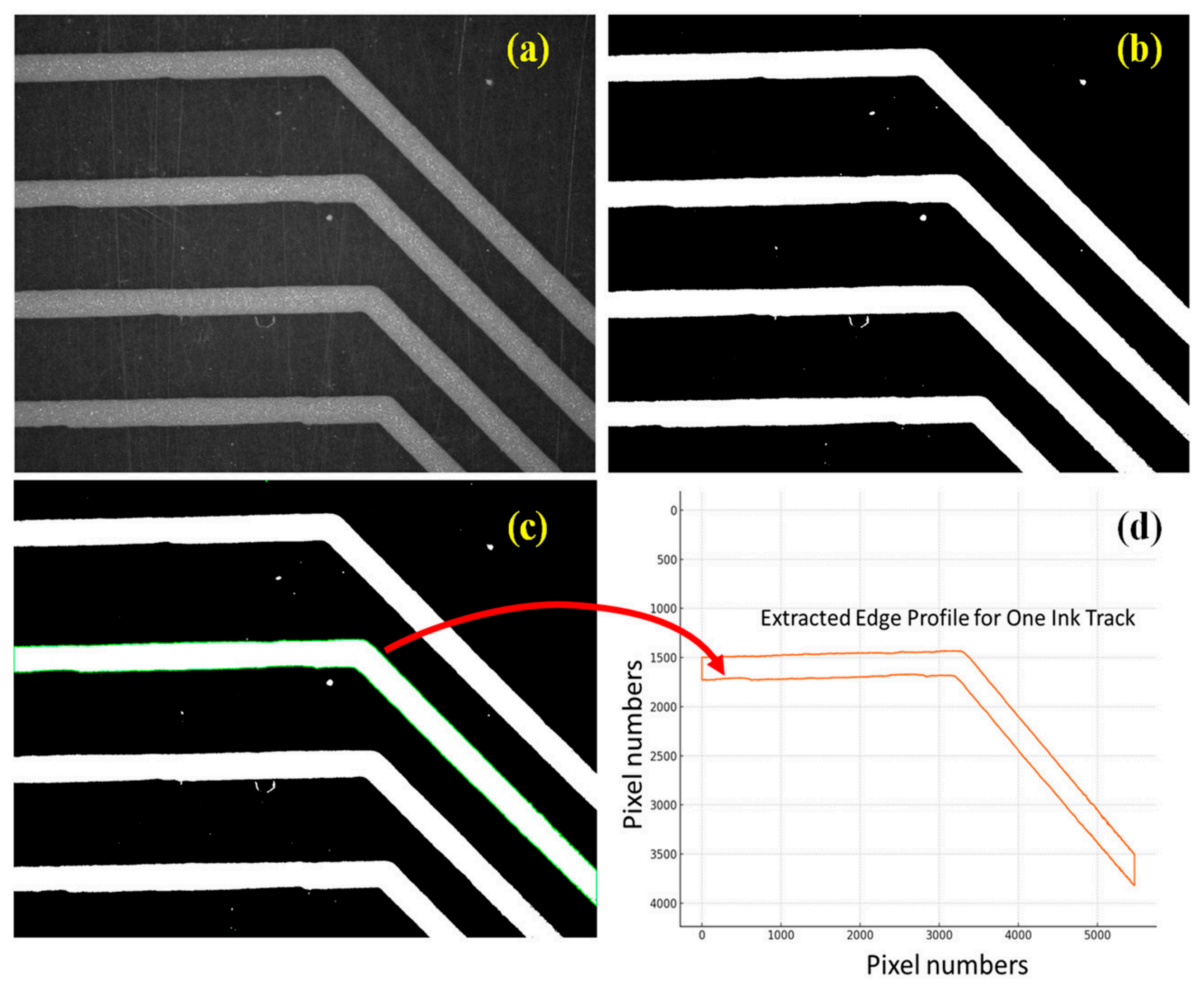
Original grayscale image (**a**), Otsu threshold image outcome (**b**), Otsu edge detection tracked line/green line (**c**), and output tracked profile line (**d**).

Complementary to segmentation, edge detection algorithms like the operator are crucial for locating sharp changes in image intensity, which often correspond to the boundaries of defects, scratches, or other surface irregularities [31,32,33] Using the Canny operator, we can calculate gradients of the image intensity in both vertical and horizontal directions, providing information about the magnitude and orientation of edges in the image. The combination of thresholding techniques like Otsu with edge detection methods like Canny can provide a robust approach for isolating and delineating defects in various imaging scenarios [16]. The Otsu edge detection outcome is illustrated as shown in Figure 5.

In this subsection, first, the principles of the Canny edge detection approach are introduced. Then, the advantages of the applied Canny operator are explained. In the horizontal (*x*) and vertical (*y*) direction, it uses two kernels obtained by convolving a simple 1D derivative with a Gaussian kernel. It works as follows: The edges in the image are identified by computing the gradient of the pixel intensities. The Canny operator computes an approximate gradient of the image intensity function. To identify the regions of conductive ink on the thin films, grayscale conversion and noise filtering were applied to the images. Otsu’s thresholding was applied to segment those regions from those on the substrate. Based on the edge detection algorithm developed by Canny, this segmented pattern was extracted. Following a four-step procedure, the Canny method enhances edge localization and robustness:

(1) Noise reduction using Gaussian smoothing, the Gaussian kernels are used before edge detection to smooth the image as in the following equation [17]:
  (7) $$G(x,y)=\frac{1}{2\pi \delta^{2}}e^{-\frac{x^{2}+y^{2}}{2\delta^{2}}}$$
  In this step, the input image *I* (*x*, *y*) is convolved with the Gaussian kernel as:
  (8) $$I_{s}(x,y)=I(x,y)*G(x,y)$$
  where σ: standard deviation of the Gaussian and ∗ is the convolution operator.
(2) Identifying gradient magnitudes and directions; at this stage, it is similar to the Sobel operator, but generally uses smoothed derivatives as in [27]:
  (9) $$G_{x}=\frac{\partial I_{s}}{\partial x}\;\;,\;\;G_{y}=\frac{\partial I_{s}}{\partial y}$$
  (10) $$\left|G\right|=\sqrt{G_{x}^{2}+G_{y}^{2}}\;\;,\;\;\theta =\tan^{-1}(\frac{G_{y}}{G_{x}})$$
(3) To thin edges, non-maximum suppression is applied; as a result, all gradient values that do not coincide with local maxima along the gradient direction are suppressed (i.e., this step typically defined algorithmically rather than as an equation).
(4) To trace edge continuity, hysteresis thresholding is used with dual thresholds by using the following thresholds that are used to trace connected edges ($T_{low}$ and $T_{high}$); there are three conditions can be applied: if ∣*G*∣ > $T_{high}$, yield to strong edge; if $T_{low}$ < ∣*G*∣ < $T_{high}$, yield to weak edge (included only if connected to strong edge); and if ∣*G*∣ < $T_{low}$ the final decision is to discard [23]. For reliable detection of conductive and identifying potential defects, gradient-based filtering was coupled with Canny edge detection. In this method, Gaussian smoothing, gradient computation, non-maximum suppression, and hysteresis thresholding are combined. This work employed a dual-threshold strategy with a lower threshold of 0.10 and an upper threshold of 0.30 (normalized intensity values). Experimentally, these threshold values were chosen to preserve edges while suppressing noise. As a result of the thresholds chosen, track boundaries could be extracted while defect-related discontinuities could be minimized, while spurious edges from illumination variations could be minimized.

**Figure 6:**
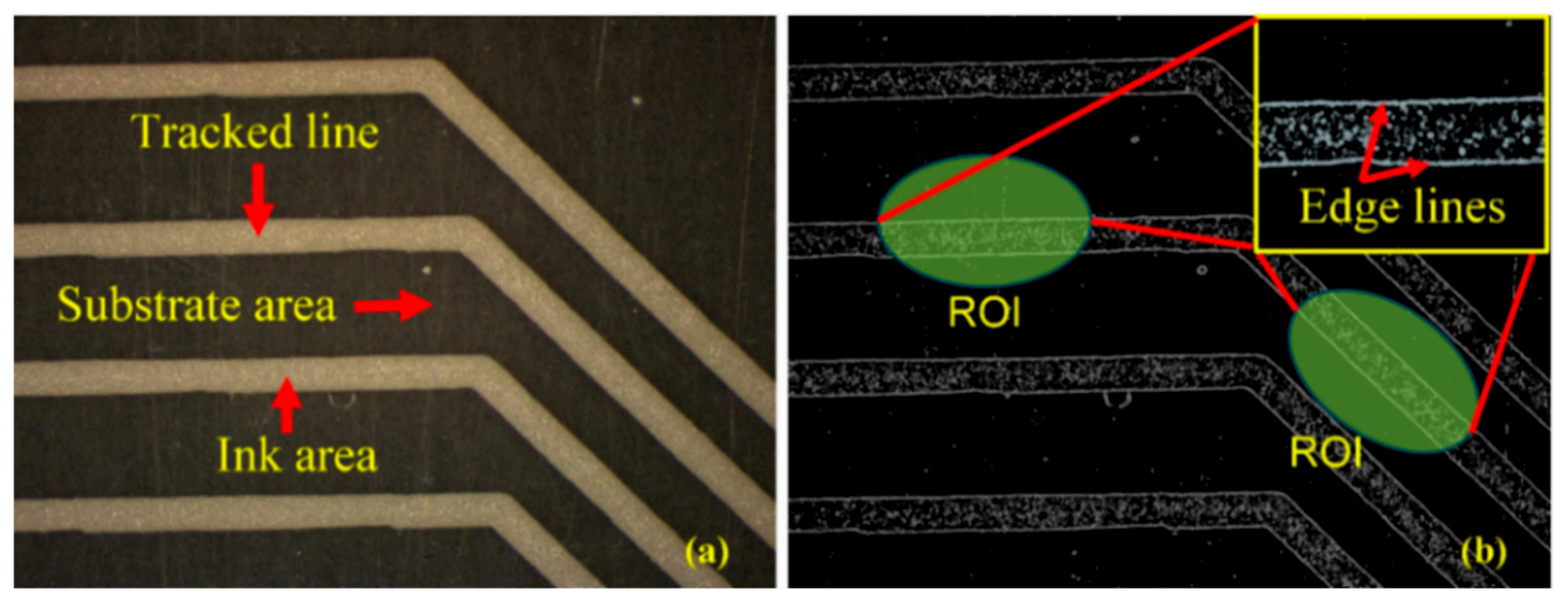
Sample tracked line (**a**), and Canny method processing result/detection edge lines (**b**).

Through this approach, boundary roughness and fine defects along the interface between the ink and substrate can be detected consistently. Canny’s algorithm produces cleaner, tighter edge profiles than traditional Sobel filtering, suitable for quantitative roughness analysis such as sample edge roughness (*Ra*) [34]. The magnitude of the gradient is indicated by (*dx*, *dy*), which is used to show the contour of the image. Surfaces with high slopes cause large gradients in the image. Coefficient 2 provides noise suppression capabilities. A simple but effective approach to approximating the gradient magnitude is to compute its *L12* norm. The *L1* norm is frequently employed in practice as a reasonable approximation of the magnitude. The specific form is $\left|\left(dx,dy\right)\right|=\left|dx\right|+\left|dy\right|$, which is significantly faster than *L2* norm. The derivation of coefficient 2 is from the quantization of the image. The Canny operator approximates horizontal and vertical gradients. All pixel values of mask are identical. The operator is selected due to the balance in gradient calculation accuracy and computational complexity [34]. Canny outcome is illustrated as shown in Figure 6.

### 4.2. Edge Sample Roughness Calculations (Ra)

**Figure 7:**
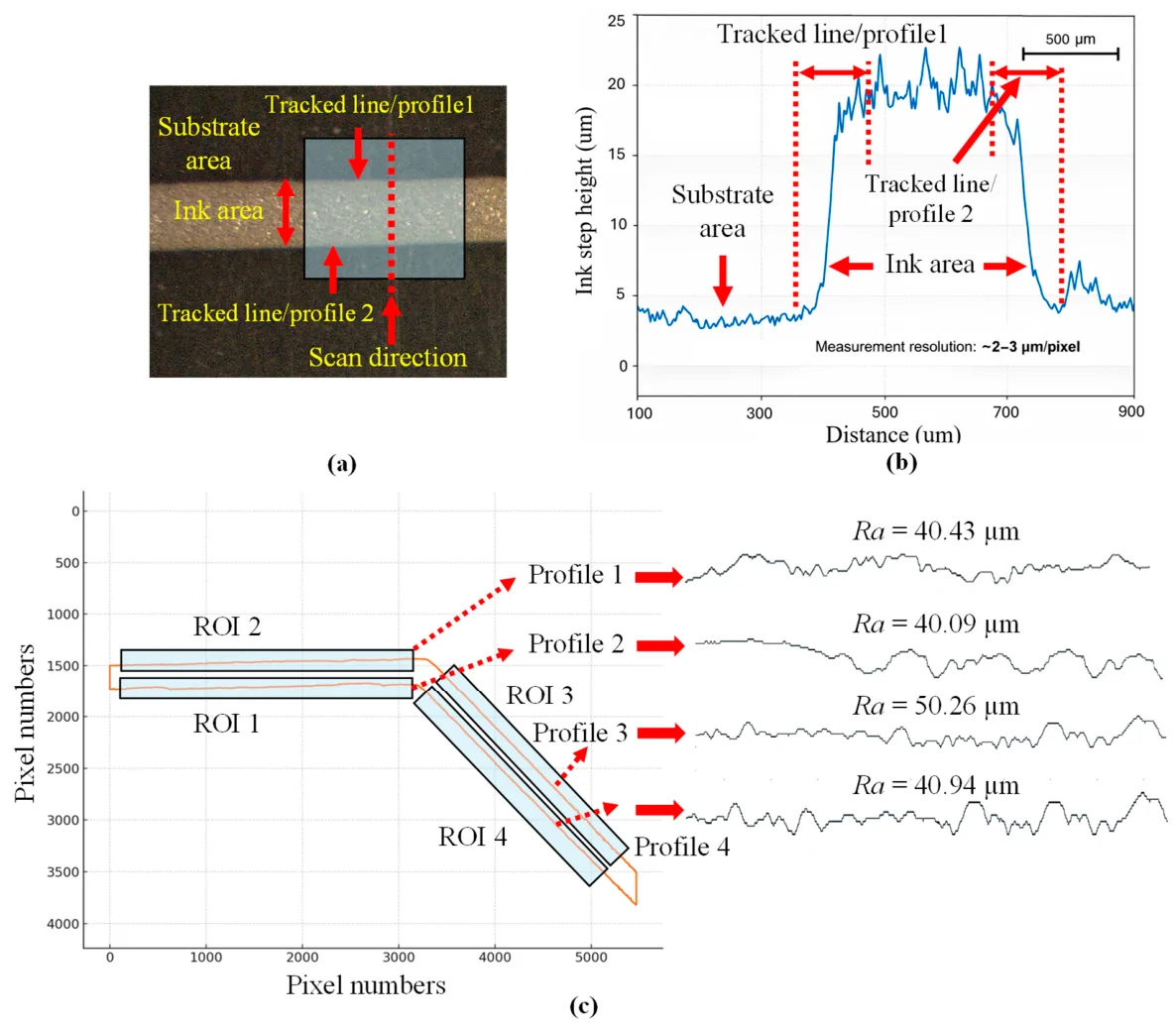
Illustrates the computed results. (**a**) Printed conductive track captured using the proposed MV system, indicating the scan direction and measurement region; (**b**) cross-sectional profile measured across the printed ink region by the tactile tool profilometer system (University of Technology); and (**c**) edge profiles extraction (profiles 1, 2, 3 and 4) using the proposed MV system along the printed track, corresponding to each ROI’s lateral edge roughness (*Ra*).

The roughness of an edge profile (ROI) is calculated after applying the Otsu method, the edge profile has been extracted based on the average absolute deviation method (*Ra*) [35]:

(11) $$Ra=\frac{1}{N}\sum_{i=1}^{N}\left|y_{i}-y^{-}\right|$$

where $y_{i}$: pixel height at edge location *i*, $y^{-}$ mean height of the edge profile and *N* is the number of edge points in the selected track. This work calculates lateral edge roughness based on the projected contour of the conductive track rather than three-dimensional surface roughness. A calibrated pixel-to-length conversion of the imaging system converts pixel coordinates into physical dimensions based on the edge profile extracted from the segmented image boundary. As a result, lateral edge roughness (*Ra*) is used in this study to describe planar contour irregularity along the edges of printed tracks. As printed electronics are made up of many geometric deviations such as ink spreading, edge waviness, and micro-defects, this method is particularly useful for evaluating their edge quality. Figure 7 shows an edge profile obtained using an MV inspection system. Spatial scale bars have been added to the figure to indicate the physical dimensions of the area under analysis. In the system, the effective spatial sampling corresponds to approximately 2–3 μm per pixel, which is sufficient for detecting small irregularities and micro-defects at the edge. Nyquist sampling criterion and optical diffraction limit dictate the measurement resolution. Several images of printed tracks were examined to evaluate measurement consistency. Based on the calibrated lateral roughness parameter (*Ra*), roughness values were calculated, and error bars were displayed where appropriate to represent statistical variation. Several edge profiles were extracted along the printed track boundaries for each region of interest. These contour deviations were used to calculate lateral roughness (*Ra*), which represents the average of the measured roughness parameters. Additionally, the standard deviation of the extracted roughness values was calculated to assess measurement stability and variability.

In contrast to the tactile profilometer, the proposed MV system performs different types of measurements. A tactile tool profilometer system generates a cross-sectional profile by scanning across the printed ink region, while MV system captures as area image and extracts lateral edge profiles along the printed ink boundary. Consequently, the measurement is used to characterize the cross-sectional structure of the printed track, while the MV measurement is used to quantify the lateral roughness (*Ra*). Figure 7 illustrates output calculations for the tracked sample profiles based on the ROI area. Under identical experimental conditions, 25 images of the printed tracks were acquired. As a result of the proposed image acquisition process, the remaining images produced comparable edge profiles. In Figure 7, we present a detailed quantitative analysis of one representative image. To verify the stability and consistency of the image acquisition process, 25 images were acquired from the same sample under identical experimental conditions. To analyse quantitative edge profile, one representative image was selected after visual inspection and consistency verification. Using this image, four regions of interest (ROIs) were selected, and each ROI was tracked and edge profiled to calculate the lateral edge roughness (*Ra*). Images that were not included in the quantitative (*Ra*) calculation were used to verify image acquisition consistency. Table 2 shows the results of using Otsu threshold computing for detecting the profiles of the sample based on the ROI areas.

Under controlled laboratory conditions, the proposed laser-enhanced MV framework was experimentally validated using silver-based conductive ink printed on a PET substrate. Image quality and edge detection performance may be affected by differences in substate type, conductive ink composition, surface reflectivity, and film thickness, even though the proposed optical imaging and image processing methodology is expected to apply to a broader range of printed electronic materials. To evaluate its robustness and suitability for R2R manufacturing environments, future work will examine the applicability of the proposed framework to alternative substrates (e.g., PI and glass), different conductive links (such as copper-based and carbon-based), and a wider range of film thanklessness.

In the current study, ambient vibration, sample reflectivity, and changes in laser speckle were not systematically evaluated on measurement performance due to controlled laboratory conditions. Ambient vibrations can affect edge localization during image acquisition by introducing small positional changes. This risk was reduced by mounting the sample on a rigid mechanical stage and keeping the imaging conditions constant. Local intensity variation caused by laser speckles can also affect image segmentation. To reduce speckle-related intensity variations, a Flat-Top Holographic Diffuser was used in the present system. The backscattered signal can also be affected by variations in sample reflectivity, which may affect the contrast between the printed track and the substrate. Under strong or non-uniform illumination changes, the current implementation uses global Otsu thresholding, which may become unsuitable. To further evaluate the robustness of the proposed system, future work will investigate adaptive thresholding and other image processing strategies, along with controlled tests of vibration, reflectivity, and speckle conditions.

## 5. Conclusions

This study describes a laser-enhanced MV system that is non-contact and measures edge profile degradation in thin film printed electronics. Otsu thresholding and Canny edge detection are integrated in the method to provide consistent segmentation and extraction of ink–substrate boundaries in high-resolution images. White light is less effective at enhancing microscale features than laser illumination, which increases contrast. Analyzing edge profile roughness (*Ra*) on multiple printed tracks produced values between 40.43 μm, 40.09 μm, 50.26 μm and 40.94 μm. The data indicates that the system can capture subtle variations in line quality and print consistency. Roll-to-roll (R2R) manufacturing inline inspection approaches were shown to be highly sensitive, fast, and scalable when applied to manufacturing. A MV approach allows non-contact inspection without destructive sampling, unlike stylus profilometry and SEM. MV frameworks are robust and efficient in providing surface metrology solutions in the next generation of flexible electronics. We will investigate real-time implementation and integration with automated process control systems. Moreover, future investigations will examine how white light illumination and alternative laser wavelengths affect defective visibility. Different types of thin film materials and defects will need to be studied in comparison to determine the optimal lighting conditions.

## Figures and Tables

**Table 1:** The literature comparison of laser and white light illumination for MV applications, included to justify the choice of laser illumination if the proposed inspection scheme.

Feature/Property	Laser Light Source	White Light Source
Image sharpness and contrast	Very high due to directional, focused beam	Moderate; depends on optics and ambient interference
Structured light projection	Ideal for line/point/plane projection in 3D vision (e.g., triangulation, profilometry)	Limited without filtering or pattern masks
3D Measurement Accuracy	High excellent for triangulation, time-of-flight, speckle-based methods	Lower due to lack of coherence and structured control
Surface Defect Detection	Effective for micro-defect detection using laser speckle, interferometry, or high-angle illumination	Suitable for macro-defect or color-based analysis

**Table 2:** Shows the output results for the tracked lines areas for the sample under test.

Sample Area	Edge Line (Profile) (μm)
Area 1 (ROI1)	40.43
Area 2 (ROI2)	40.09
Area 3 (ROI3)	50.26
Area 4 (ROI4)	40.94

## Data Availability

The data that support the findings of this study are available from the corresponding author upon reasonable request.
